# Surgery of the Lungs and Pleura

**Published:** 1894-09-08

**Authors:** 


### SURGERY OF THE LUNGS AND PLEURA.

**Empyæma.**—The question whether it is desirable in all cases to resect a piece of rib in opening an empyæma is discussed by Dr. Carl Beck, of New York[1]. He is strongly of opinion that it is necessary, as again and again he says he has found "clots consisting of fibrinous or cheesy products attached to the pleura," and he considers that they can only be satisfactorily removed by introducing the finger into the cavity and turning them out, and that in order to do this a large opening should be made by resection of a portion of the rib. His experience has been that in nine out of ten cases these fibrinous clots would be present, free in the cavity as well as adherent to the pleura. An attempt was made to dissolve them by the injection of pepsine, but without avail. Dr. Beck rarely uses a general anæsthetic, but performs the operation under ether spray, as he considers the danger of the anæsthetic with one side of the chest full of fluid very great. On this point the results of anæsthesia at the Liverpool Children's Hospital, to which we shall presently refer, may be noted. He resects the sixth rib, between the anterior and posterior axillary lines, and he points out what is undoubtedly an advantage of this situation, that the patient can lie on his back and need not turn on his side, and thus impair the movement of the sound lung during the anæsthesia. Another precaution to which he calls attention is also an important one—to press a sponge against the opening from time to time to interrupt the stream, and thus avoid a too rapid expansion of the lungs. Dr. Beck uses an irrigation of bichloride solution, "mainly for mechanical purposes, that is, to evacuate thoroughly." This is, of course, contrary to the custom in this country, which is only to wash out the pleural cavity when the pus is offensive, and we shall presently refer to a paper by Dr. Fisher reminding us of its dangers. A suggestion of Dr. Beck's to stitch the edge of the pleural opening to the skin is a good one, as it allows of easy reintroduction of the tube.

At the American Surgical Association, Dr. John Ashhurst, jun., of Philadelphia,[2] read a paper on the "Surgical Treatment of Empyæma." He considered that no operation was justifiable unless the presence of pus was certain, or unless thorough treatment by medicinal agents, blisters, &c., had failed, or unless dyspnoea was urgent. But with the exploring syringe at hand we need not long be in doubt as to the presence of pus in most cases, and when found the sooner it is treated surgically the better, as was recommended by Dr. Nancrede in the discussion which followed the reading of Dr. Ashhurst's paper. Dr. Ashhurst ad vised that the first operation should consist of aspiration only, but as Mr. Godlee showed in a paper, referred to in The Hospital, in May (page 123), this seldom succeeds, and it is better, as Dr. Nancrede remarked, to provide for the permanent evacuation of the pus immediately. Dr. Prewitt stated that he had seen several recoveries follow aspiration in children, but never in adults. We, in this country, who are accustomed to cut our drainage tube so short that it only just reaches the pleural cavity, as Godlee advises, may be rather surprised to read of Dr. Ashhurst's proposal to make two openings, and carry a large drainage tube through the plural cavity from one opening to the other.

Dr. Demosthen and Dr. Calinescu, of Bucharest,[3] record thirty-seven cases of empyæma treated surgically, and, in opposition to Dr. Ashhurst, they state that they never saw a case of empyæma cured by medical means; and they urge the practice of surgical measures in all cases of suppurative pleurisy, and the sooner the better, for in their experience the results were always the more striking the nearer the onset of the disease the treatment was instituted. They consider that as a rule recovery is slow, but in some cases surgical measures were followed by a "veritable resurrection, and from poor emaciated, cachectic subjects the patients became rapidly fat and healthy-looking."

A new operation for the cure of chronic empyæma was brought before the notice of the French Congress of Surgery in 1893 by M. Delorne, and last January he had an opportunity of performing the operation for the first time[4]. He removes the false membrane binding down the lung, instead of resecting the chest-wall, and thus allows the lung to expand, rather than the chest-wall to fall in. A large trap-door was opened in the wall of the chest by dividing the ribs in front and half cutting them through behind, and then fracturing them, turning back the entire flap with the soft parts attached to the ribs. The parietal and visceral pleura were covered with a thick granular false membrane. After scraping away the fungus granulations, the position of the heart and lung under the thick layer of the pleura could not be made out, the operation being performed on the left side. The upper part of the visceral pleura was then incised, and the lung beneath discovered. He introduced a sound and then his finger between the lung and thickened pleura, so as to detach them, and through this opening the lung protruded, on the patient coughing, beyond the level of the thoracic-wall, so that it was necessary to push it back in order to continue the operation. After further separation of the thickened pleura, the trap-door was closed. The patient was progressing favourably when the case was reported.

Delagénière[5] considers binding down of the lung by thickened pleura is not so frequent as is generally supposed, but that the non-closure of pleural fistulæ is due to want of sufficient drainage of what he calls the costo-diaphragmatic cul-de-sac, between the sixth, seventh, and eighth ribs and the diaphragm, and he practices almost complete removal of these three ribs, which is practically Estlander's operation.

Dr. Theodore Fisher[6] records a case in which, on pumping air charged with eucalyptus vapour into the pleural cavity for the treatment of empyæma, the patient became quite suddenly semi-unconscious, and pulse and respiration failed for a time. Hemiplegia on the side opposite to the empyæma was present, but a recovery followed. Dr. Fisher discusses the possible cause of such symptoms every now and then recorded as occurring during the washing out of an empyæma, and suggests that the coldness of the air in his case, and of the fluid in other cases, may have been the cause, and suggests that the fluid used should always be at body temperature.

The treatment of double empyæma in children and young adults is discussed by Dr. Sutherland,[10] and several cases reported. He considers that some special precautions are certainly necessary, for if both sides are evacuated at the same time by incision, cardiac and respiratory failures, as might be expected, have frequently been alarming. Many of the cases are localised empyæmata, and here the difficulty does not arise. Aspiration, as recommended by Mr. Godlee, Dr. Sutherland proposes to employ for one side until the lung on the other side has time to expand, incising the side which contains the largest amount of pus. Of twenty-one cases, the records of which Dr. Sutherland has tabulated, only one died. Of course, many unpublished cases have died, but this shows that the mortality is at any rate less than one might suppose. The results in these twenty-one published cases show that aspiration as a means of treatment other than palliative is useless, and in one case aspiration was performed eleven times in the course of six months without improvement in the condition of the patient, but double resection with drainage was successful. In another aspiration was performed five times, and it was not until an incision was made that the patient began to mend. So rapid was the reaccumulation of fluid after aspiration that in one case eight pints of pus were removed in the course of five aspirations.

The results of treatment of one hundred and twenty cases of empyæma at the Liverpool Children's Hospital are given by Mr. Wightman, late house surgeon there.[11] He concludes that aspiration for empyæma as routine practice in preliminary treatment should be condemned; that chloroform may be safely administered, only one death from anæsthesia occurring in the large number of cases treated, and this occurred where the child was almost moribund when the treatment was begun; and that incision and drainage were found in the majority of cases quite sufficient. Dr. Batten[12] considers that better results can be obtained, however, by resection of a portion of a rib, and he gives a table showing a smaller mortality after such treatment than after simple incision and drainage, as shown in Mr. Wightman's table. He says at the Great Ormond Street Children's Hospital resection is the treatment always adopted, and his tables are of cases treated at that hospital.

[1] Medical Record, May 19, 1894. [2] Ibid, June 2, 1894, and New York Med. Journal, July 7, page 23, 1894. [3] Medical Record, p. 570, May 5, 1894. [4] L'Union Médicale, Jan. 25, 1894, and The Quarterly Medical Journal, April, 1894, p. 261, and Therep. Gaz., March 15, 1894, p. 197. [5] Archives Provinciales de Chirurgie, 1894; and Brit. Med. Journal, April 21, 1894. [6] Lancet, March 17, p. 668, 1894. [7] Siglo, Medico, April, 1894; and Brit. Med. Journ., April 28, p. 65. [8] Journal des Praticiens, Feb. 21, 1894; aad Lancet, March 17, p. 693. [9] Lancet, June 9, 1894, p. 1469. [11] Ibid, May 5, 1894, p. 1128. [12] Ibid, June 2, 1894, p. 1[illegible]68.

